# Genome-wide screening of the genes required for tolerance to vanillin, which is a potential inhibitor of bioethanol fermentation, in *Saccharomyces cerevisiae*

**DOI:** 10.1186/1754-6834-1-3

**Published:** 2008-04-15

**Authors:** Ayako Endo, Toshihide Nakamura, Akira Ando, Ken Tokuyasu, Jun Shima

**Affiliations:** 1 National Food Research Institute, 2-1-12 Kannondai, Tsukuba, Ibaraki 305-8642, Japan

## Abstract

**Background:**

Lignocellulosic materials are abundant and among the most important potential sources for bioethanol production. Although the pretreatment of lignocellulose is necessary for efficient saccharification and fermentation, numerous by-products, including furan derivatives, weak acids, and phenolic compounds, are generated in the pretreatment step. Many of these components inhibit the growth and fermentation of yeast. In particular, vanillin is one of the most effective inhibitors in lignocellulose hydrolysates because it inhibits fermentation at very low concentrations. To identify the genes required for tolerance to vanillin, we screened a set of diploid yeast deletion mutants, which are powerful tools for clarifying the function of particular genes.

**Results:**

Seventy-six deletion mutants were identified as vanillin-sensitive mutants. The numerous deleted genes in the vanillin-sensitive mutants were classified under the functional categories for 'chromatin remodeling' and 'vesicle transport', suggesting that these functions are important for vanillin tolerance. The cross-sensitivity of the vanillin-sensitive mutants to furan derivatives, weak acids, and phenolic compounds was also examined. Genes for ergosterol biosynthesis were required for tolerance to all inhibitory compounds tested, suggesting that ergosterol is a key component of tolerance to various inhibitors.

**Conclusion:**

Our analysis predicts that vanillin tolerance in *Saccharomyces cerevisiae *is affected by various complicated processes that take place on both the molecular and the cellular level. In addition, the ergosterol biosynthetic process is important for achieving a tolerance to various inhibitors. Our findings provide a biotechnological basis for the molecular engineering as well as for screening of more robust yeast strains that may potentially be useful in bioethanol fermentation.

## Background

The production and utilization of bioethanol as an alternative fossil fuel have attracted attention in the effort to prevent global warming and improve energy reserves [1,2]. Bioethanol production generally utilizes derivatives from food crops such as corn grain and sugarcane, but the limited supply of these crops can lead to competition between their use in bioethanol production and food provision. Lignocellulosic materials such as crop residues and wood chips are among the most important potential sources for bioethanol production [3,4].

Lignocellulosic plant residue contains up to 70% carbohydrates (as cellulose and hemicellulose), so is a prominent substrate for inexpensive bioethanol production [5]. However, due to the close association of cellulose and hemicellulose with lignin in the plant cell wall, pretreatment is necessary to make carbohydrates available for enzymatic hydrolysis and fermentation [6]. For economic reasons, dilute acid hydrolysis is commonly used to prepare lignocelluloses for enzymatic saccharification and fermentation [7]. Numerous by-products, including furan derivatives, weak acids, and phenolic compounds, are generated during pretreatment. It has been suggested that many of these components inhibit the growth and fermentation of yeast [8-10]. Furan derivatives such as furfural and 5-hydroxymethylfurfural (HMF), which are generated by the breakdown of sugars, have been reported to be fermentation inhibitors [11]. Furfural and/or HMF have been shown to reduce enzymatic and biological activity and breakdown of DNA [12,13]. Phenolic compounds such as vanillin and 4-hydroxybenzoic acid (HB), generated by lignin degradation, have also been shown to be potent fermentation inhibitors [11]. In particular, vanillin has been suggested as a more effective inhibitor of growth and bioethanol fermentation than the furan derivatives, weak acids, and other phenolic compounds, because vanillin inhibits fermentation at low concentrations [11]. In *Saccharomyces cerevisiae*, vanillin is likely to be converted to vanillyl alcohol and vanillic acid by oxidoreductase enzyme(s) [14], and a mutant of the mitochondrial superoxide dismutase (Mn-SOD) gene (*SOD2*) was shown to exhibit enhanced vanillin-induced growth inhibition [15]. However, to date there have been few studies of the genes required for conferring tolerance to vanillin in *S. cerevisiae *[15].

Collections of yeast deletion mutants can be powerful tools: the function of particular genes can be clarified by analyzing the phenotypes of mutants lacking genes of interest; this is known as 'phenomics' [16-20]. An international consortium has carried out the systematic deletion of all of the open reading frames of *S. cerevisiae *by using a polymerase chain reaction (PCR)-mediated gene deletion strategy [16]. Recently, more than 62 genes were found to be associated with a sensitivity to furfural using a *S. cerevisiae *disruption library [21]. These results served as the basis for a model of the furfural conversion pathway [22].

In this study, we screened the yeast deletion mutant collection to identify the genes required for tolerance to vanillin in *S. cerevisiae*. Moreover, the cross-sensitivity of the vanillin-sensitive mutants to other inhibitors (for example, furan derivatives, weak acids, and phenolic compounds) was examined. Here, we discuss the mode of inhibition induced by vanillin and the design of a more robust strain of *S. cerevisiae *to increase the efficiency of bioethanol fermentation.

## Results and discussion

### Determination of experimental conditions for screening of vanillin-sensitive strains

**Figure 1:**
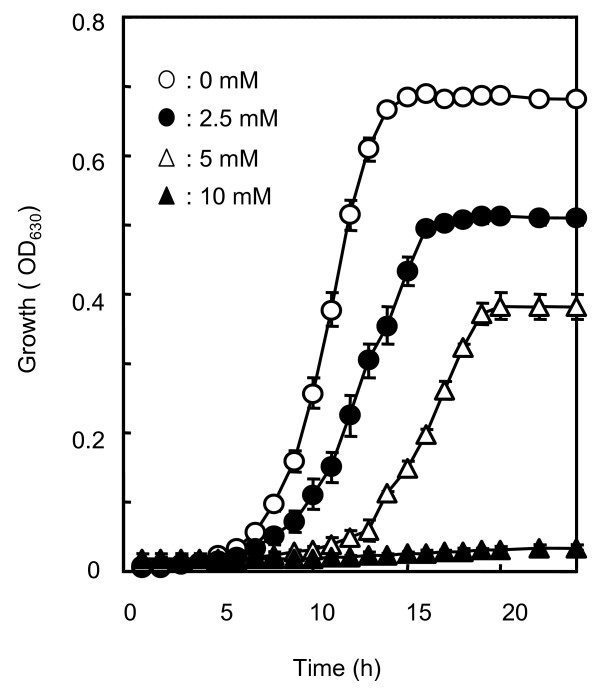
**The effects of vanillin addition on the growth of *S. cerevisiae *BY4743**. Cells were grown in YPD medium supplemented with vanillin at various concentrations, and cell growth was monitored by measurement of optical density at 630 nm. Values are expressed as mean ± standard deviation from triplicate experiments.

To determine suitable experimental conditions for the evaluation of the sensitivity of the mutants to vanillin, wild-type strain BY4743 was cultivated in YPD media containing 0-10 mM vanillin. Figure 1 shows the growth curves with various vanillin concentrations. At a vanillin concentration of 5 mM, growth of the wild-type strain was inhibited by approximately 50%. The growth inhibition rate of strains exposed to vanillin was correlated with ethanol productivity (data not shown). Based on these results, we selected YPD medium containing 5 mM vanillin for further analysis, and the growth of the strains was measured after incubation for 24 hours at 30°C to screen for vanillin-sensitive mutants.

### Screening of genes required for tolerance to vanillin

**Figure 2:**
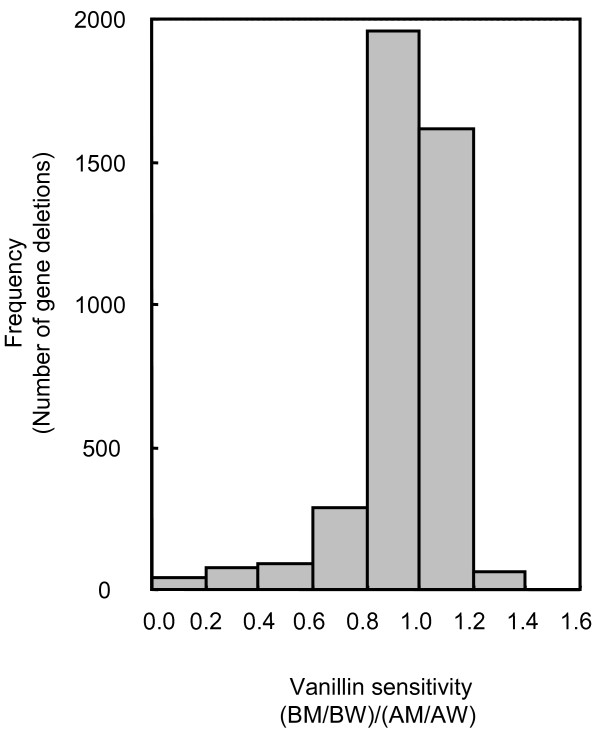
**Frequency distribution of vanillin sensitivity ofgene-deletion mutants**. Vanillin sensitivity is expressed as (BM/BW)/(AM/AW). Mutants with (BM/BW)/(AM/AW) values less than 0.3 were defined as vanillin-sensitive strains.

To identify vanillin-sensitive mutants, we screened the complete mutant collection consisting of approximately 4700 diploid deletion strains. Prior to analysis of the data to determine vanillin sensitivity, mutants that exhibited considerable growth inhibition without vanillin treatment were omitted (see methods). We then examined the vanillin sensitivity of 4127 deletion mutants. The distribution of the deletion-mutant vanillin sensitivity parameter was presented in a frequency distribution plot in order to obtain an overall view of the effects of mutants on vanillin sensitivity (Fig. 2). Among the 4127 deletion mutants, we defined 76 strains (approximately 1.8% of all strains) in which vanillin tolerance was less than 30% of that of the wild-type strain. The genes deleted in these 76 mutants were considered to be those required for tolerance to vanillin.

**Figure 3:**
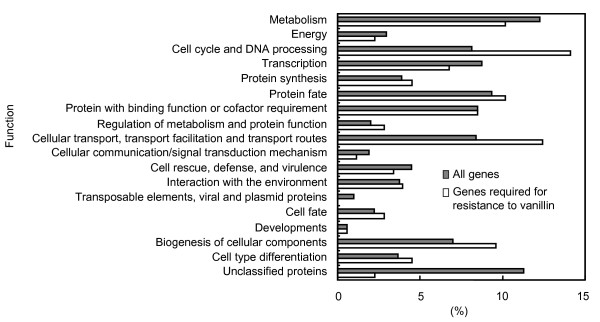
**Functional categorization of genes required for tolerance to vanillin (total number, 76) and of all genes in *S. cerevisiae***. Classifications were performed based on the categories defined in the MIPS database using FunCat [31].

Sensitivity to vanillin was yielded by deletion of the genes listed in Additional file 1, which also provides a tentative organization of the genes according to functional categories defined in the Munich Information Center for Protein Sequences (MIPS) database (note that single genes are frequently placed under several different functional categories in this database). Figure 3 shows the results of the classification of the deleted genes based on the functional category listed in the MIPS database. This categorization helped to clarify the gene functions involved in the tolerance to vanillin; in other words, the functional categories that contain a high number of genes are thought to represent cellular functions that are important for conferring the tolerance to vanillin. The results of this categorization revealed that those deletions capable of conferring some degree of vanillin sensitivity upon strains were more frequently (more than 1.5-fold) included under the 'cell cycle and DNA processing' and 'cellular transport, transport facilitation, and transport routes' categories.

The 'cell cycle and DNA processing' category contained a high number of genes involved in 'chromatin remodeling' (11 out of 17 genes, Additional file 1). It has been reported that the genes in this group were required for tolerance to DNA-damaging agents such as ionizing radiation in *S*. *cerevisiae *[23]. Among the types of damage caused by ionizing radiation, the most biologically relevant lesions are DNA double-strand breaks (DSBs). It is known that vanillin blocks DNA end-joining, which is a major pathway of DSB repair in mammalian cells, by directly inhibiting the activity of DNA-dependent protein kinase, a crucial non-homologous DNA end-joining enzyme [24]. Based on this information, we speculated that vanillin might cause serious DNA damage via two processes, that is, DNA breakdown and during the subsequent DNA repair process.

The 'cellular transport, transport facilitation, and transport routes' category frequently included genes involved in 'vesicle transport' (12 out of 18 genes; see Additional file 1). Moreover, 7 of the 12 genes involved in 'vesicle transport' were required for rapamycin tolerance [25]. Whether the inhibitory mechanisms of vanillin were similar to those of rapamycin remains uncertain.

### Cross-sensitivity of vanillin-sensitive mutants to furan derivatives, weak acids, and other phenolic compounds

Because the toxicity of inhibitors is enhanced by a combination of different inhibitory compounds [26], the overlap between a sensitivity to vanillin and to other inhibitors (that is, furan derivatives, weak acids, and phenolic compounds) was assessed by examining the cross-sensitivities of the mutants identified as being vanillin-sensitive. To determine suitable measurement conditions, we cultivated BY4743 cells according to the same method as used in the assessment of vanillin sensitivity. We determined the inhibitor concentration (that is, 50% inhibition of growth of the wild-type strain), and cross-sensitivity was assessed under the conditions determined as above (data not shown, see methods). Additional file 2 shows the sensitivity of all of the vanillin-sensitive mutants to various inhibitors. We defined the genes required for tolerance to each inhibitor; the threshold for sensitivity was less than 0.3 (Additional file 2). To identify correlations between gene function and the chemical structure of the inhibitors, the functional distributions of genes involved in conferring tolerance to each inhibitor were categorized using the MIPS database (data not shown). There did not appear to be any strong correlations between chemical structure and gene function required for tolerance, because the genes involved in tolerance to each inhibitor were not classified into specific categories depending on chemical structures.

We found that the genes required for tolerance to all inhibitors were significantly classified under the subcategory 'tetracyclic and pentacyclic triterpene (cholesterin, steroids, and hopanoids) metabolism', which is a subcategory under 'metabolism'. The *p *value, which represented the probability that the intersection of a given list with any given functional category would occur by chance, for each inhibitor was less than 0.001. This subcategory contained genes encoding enzymes involved in the ergosterol biosynthetic process (for example, *ERG3*, *ERG6*, *ERG2 *and *ERG24*). These findings strongly suggest that ergosterol, which is a ubiquitous component of cellular membranes in yeast, is required for the acquisition of tolerance to all of the inhibitors tested here. Because ergosterol is transported throughout the cell via vesicular and non-vesicular trafficking mechanisms [27], it is reasonable to speculate that the sensitivity of vesicle transport-deficient mutants to various inhibitors may be the result of defects in the delivery of ergosterol to the proper location(s). Recently, Aguilera et al. reported finding a correlation between ergosterol and ethanol tolerance [28]. Taken together, these results suggest that certain bioengineering approaches to ergosterol biosynthesis such as molecular breeding of yeast strains with a high ergosterol content can overcome the limitations of bioethanol production that are a result of the inhibitory effects on yeast growth by the by-products of lignocellulose hydrolysis and ethanol. Ergosterol may also serve as a marker in screening for strains that are suitable for bioethanol fermentation.

## Conclusion

In this study, we successfully identified genes involved in vanillin tolerance. These genes were classified as being involved in a wide range of cellular processes, in particular, in chromatin remodeling and vesicle transport. We speculate that vanillin tolerance in *S. cerevisiae *is affected by various complicated processes that take place on both the molecular and the cellular level. The results of cross-sensitivity of the vanillin-sensitive mutants to other inhibitors indicated that the ergosterol biosynthetic process was important for achieving tolerance to various inhibitors. The data obtained in this study are expected to be useful for molecular engineering as well as for screening for more robust strains that may potentially be useful in bioethanol fermentation.

## Methods

### Yeast strains and media

*Saccharomyces cerevisiae *BY4743 (*MAT***a**/α *his3Δ1/his3Δ1 leu2Δ0/leu2Δ0 lys2Δ0/LYS2 MET15/met15Δ0 ura3Δ0/ura3Δ0*) and the complete collection of diploid deletion strains (*MAT****a****/α*) constructed by the insertion of *kanMX4 *cassettes (geneticin resistance) as selective markers to the genome of BY4743 [16] were obtained from EUROSCARF (European *Saccharomyces cerevisiae *archive for functional analysis).

Yeast cells were grown at 30°C on solid and in liquid YPD medium consisting of 10 g of yeast extract (Difco laboratories, Detroit, MI, USA), 20 g of peptone (Difco) and 20 g of glucose (per liter). Deletion mutant strains were maintained on YPD agar supplemented with 200 μg/ml of geneticin (Sigma-Aldrich, St Louis, MO, USA). Sensitivity to vanillin was assessed using YPD containing 5 mM vanillin (Nacalai Tesque, Kyoto, Japan).

### Identification of gene deletions yielding sensitivity to vanillin

Deletion mutant and wild-type strains were inoculated into 100 μl YPD liquid medium in microtiter plates (Corning Inc, Corning, NY, USA) using a 48-pin replicator (Funakoshi, Tokyo, Japan) and then the inoculates were cultivated at 30°C for 48 hours (preculture). Using the 48-pin replicator, portions (1.2 μl) of the preculture were transferred into 100 μl YPD and YPD containing 5 mM vanillin, and then the cultures were incubated for 24 hours at 30°C. The optical density at 630 nm (OD_630_) of the cultures was measured using a microtiter-plate reader (Elx800, BioTek, Winooski, VT, USA). To maximize standardization and reproducibility for each run, the quantitative growth data measured in each microtiter plate were normalized to the average response of three replicates of the wild-type strain (BY4743) included in each run.

Four parameters were used in the data analysis of the sensitivity to vanillin in the deletion mutants: (1) AM, defined as the OD_630 _of mutant strains in YPD medium, (2)BM, defined as the OD_630 _of mutant strains in YPD with 5 mM vanillin, (3) AW, defined as the OD_630 _of BY4743 (wild-type strain) in YPD medium, and (4) BW, defined as the OD_630 _of BY4743 in YPD with 5 mM vanillin. Mutants that exhibited considerable growth inhibition in YPD medium (that is, AM/AW < 0.9) were excluded from further data analysis. The value of (BM/BW)/(AM/AW) was used as the parameter for sensitivity to vanillin. In this calculation, AM/AW values were used to compensate for growth defects in samples without vanillin treatment.

### Classification of genes

Genes were assigned to functional categories using the MIPS database [29] and the *Saccharomyces *Genome Database [30].

## Competing interests

The authors declare that they have no competing interests.

## Authors' contributions

AE carried out all of the experiments, participated in the study design and wrote the manuscript. TN participated in the design of the study and helped in writing. AA contributed to the study design, data analysis and manuscript writing. KT contributed to the manuscript writing. JS directed the overall study and the manuscript writing. All authors read and approved the final manuscript.

## Supplementary Material

Additional file 1 Gene deletions associated with sensitivity to vanillin. This list shows the vanillin sensitivity of the deletion mutants. Click here for file

Additional file 2 Cross-sensitivity of vanillin-sensitive mutants to various inhibitors. This list shows the cross-sensitivity of the vanillin-sensitive mutants to various fermentation inhibitors. Click here for file

## References

[B1] Lin Y, Tanaka S (2006). Ethanol fermentation from biomass resources: current state and prospects. Appl Microbiol Biotechnol.

[B2] Vertes AA, Inui M, Yukawa H (2006). Implementing biofuels on a global scale. Nat Biotechnol.

[B3] Hahn-Hagerdal B, Galbe M, Gorwa-Grauslund MF, Liden G, Zacchi G (2006). Bio-ethanol - the fuel of tomorrow from the residues of today. Trends Biotechnol.

[B4] Gray KA, Zhao L, Emptage M (2006). Bioethanol. Curr Opin Chem Biol.

[B5] Zaldivar J, Nielsen J, Olsson L (2001). Fuel ethanol production from lignocellulose: a challenge for metabolic engineering and process integration. Appl Microbiol Biotechnol.

[B6] McMillan JD, Himmel ME, Baker JO, Overend RP (1994). Pretreatment of lignocellulosic biomass. Enzymatic Conversion of Biomass for Fuels Production (American Chemical Society Symposium Series).

[B7] Galbe M, Zacchi G (2002). A review of the production of ethanol from softwood. Appl Microbiol Biotechnol.

[B8] Olsson L, Hahn-Hagerdal B (1996). Fermentation of lignocellulosic hydrolysates for ethanol production. Enzyme Microb Technol.

[B9] Palmqvist E, Hahn-Hagerdal B (2000). Fermentation of lignocellulosic hydrolysates. II: inhibitors and mechanisms of inhibition. Bioresour Technol.

[B10] Saha BC (2003). Hemicellulose bioconversion. J Ind Microbiol Biotechnol.

[B11] Klinke HB, Thomsen AB, Ahring BK (2004). Inhibition of ethanol-producing yeast and bacteria by degradation products produced during pre-treatment of biomass. Appl Microbiol Biotechnol.

[B12] Modig T, Liden G, Taherzadeh MJ (2002). Inhibition effects of furfural on alcohol dehydrogenase, aldehyde dehydrogenase and pyruvate dehydrogenase. Biochem J.

[B13] Khan QA, Hadi SM (1994). Inactivation and repair of bacteriophage lambda by furfural. Biochem Mol Biol Int.

[B14] Fitzgerald DJ, Stratford M, Narbad A (2003). Analysis of the inhibition of food spoilage yeasts by vanillin. Int J Food Microbiol.

[B15] Kim JH, Campbell BC, Mahoney N, Chan KL, May GS (2006). Targeting antioxidative signal transduction and stress response system: control of pathogenic *Aspergillus *with phenolics that inhibit mitochondrial function. J Appl Microbiol.

[B16] Giaever G, Chu AM, Ni L, Connelly C, Riles L, Veronneau S, Dow S, Lucau-Danila A, Anderson K, Andre B, Arkin AP, Astromoff A, El-Bakkoury M, Bangham R, Benito R, Brachat S, Campanaro S, Curtiss M, Davis K, Deutschbauer A, Entian KD, Flaherty P, Foury F, Garfinkel DJ, Gerstein M, Gotte D, Guldener U, Hegemann JH, Hempel S, Herman Z, Jaramillo DF, Kelly DE, Kelly SL, Kotter P, LaBonte D, Lamb DC, Lan N, Liang H, Liao H, Liu L, Luo C, Lussier M, Mao R, Menard P, Ooi SL, Revuelta JL, Roberts CJ, Rose M, Ross-Macdonald P, Scherens B, Schimmack G, Shafer B, Shoemaker DD, Sookhai-Mahadeo S, Storms RK, Strathern JN, Valle G, Voet M, Volckaert G, Wang CY, Ward TR, Wilhelmy J, Winzeler EA, Yang Y, Yen G, Youngman E, Yu K, Bussey H, Boeke JD, Snyder M, Philippsen P, Davis RW, Johnston M (2002). Functional profiling of the *Saccharomyces cerevisiae *genome. Nature.

[B17] Warringer J, Ericson E, Fernandez L, Nerman O, Blomberg A (2003). High-resolution yeast phenomics resolves different physiological features in the saline response. Proc Natl Acad Sci USA.

[B18] Fernandez-Ricaud L, Warringer J, Ericson E, Pylvanainen I, Kemp GJ, Nerman O, Blomberg A (2005). PROPHECY - a database for high-resolution phenomics. Nucleic Acids Res.

[B19] Ando A, Tanaka F, Murata Y, Takagi H, Shima J (2006). Identification and classification of genes required for tolerance to high-sucrose stress revealed by genome-wide screening of *Saccharomyces cerevisiae*. FEMS Yeast Res.

[B20] Ando A, Nakamura T, Murata Y, Takagi H, Shima J (2007). Identification and classification of genes required for tolerance to freeze-thaw stress revealed by genome-wide screening of *Saccharomyces cerevisiae *deletion strains. FEMS Yeast Res.

[B21] Gorsich SW, Dien BS, Nichols NN, Slininger PJ, Liu ZL, Skory CD (2006). Tolerance to furfural-induced stress is associated with pentose phosphate pathway genes *ZWF1*, *GND1*, *RPE1*, and *TKL1 *in *Saccharomyces cerevisiae*. Appl Microbiol Biotechnol.

[B22] Liu ZL (2006). Genomic adaptation of ethanologenic yeast to biomass conversion inhibitors. Appl Microbiol Biotechnol.

[B23] Bennett CB, Lewis LK, Karthikeyan G, Lobachev KS, Jin YH, Sterling JF, Snipe JR, Resnick MA (2001). Genes required for ionizing radiation resistance in yeast. Nat Genet.

[B24] Durant S, Karran P (2003). Vanillins - a novel family of DNA-PK inhibitors. Nucleic Acids Res.

[B25] Xie MW, Jin F, Hwang H, Hwang S, Anand V, Duncan MC, Huang J (2005). Insights into TOR function and rapamycin response: chemical genomic profiling by using a high-density cell array method. Proc Natl Acad Sci USA.

[B26] Oliva JM, Ballesteros I, Negro MJ, Manzanares P, Cabanas A, Ballesteros M (2004). Effect of binary combinations of selected toxic compounds on growth and fermentation of *Kluyveromyces marxianus*. Biotechnol Prog.

[B27] Schulz TA, Prinz WA (2007). Sterol transport in yeast and the oxysterol binding protein homologue (OSH) family. Biochim Biophys Acta.

[B28] Aguilera F, Peinado RA, Millan C, Ortega JM, Mauricio JC (2006). Relationship between ethanol tolerance, H^+ ^-ATPase activity and the lipid composition of the plasma membrane in different wine yeast strains. Int J Food Microbiol.

[B29] Munich Information Center for Protein Sequences (MIPS). http://mips.gsf.de/.

[B30] *Saccharomyces *Genome Database (SGD). http://www.yeastgenome.org/.

[B31] Ruepp A, Zollner A, Maier D, Albermann K, Hani J, Mokrejs M, Tetko I, Guldener U, Mannhaupt G, Munsterkotter M, Mewes HW (2004). The FunCat, a functional annotation scheme for systematic classification of proteins from whole genomes. Nucleic Acids Res.

