# Supplementary material for: Genome-wide screening of the genes required for tolerance to vanillin, which is a potential inhibitor of bioethanol fermentation, in Saccharomyces cerevisiae
Source: Biotechnol Biofuels. 2008 Apr 15;1:3. doi: 10.1186/1754-6834-1-3 (PMC2375868; doi:10.1186/1754-6834-1-3)
Supplement: Additional file 1 — Gene deletions associated with sensitivity to vanillin. This list shows the vanillin sensitivity of the deletion mutants. [file 1754-6834-1-3-S1.doc]

## Additional file 1 - Gene deletions associated with sensitivity to vanillin

|  | ORF | Gene | Sensitivity^a^ | Description |
| --- | --- | --- | --- | --- |
| Metabolism | | | | |
|  | YML115C | *VAN1* | 0.25 | Vanadate resistance protein |
|  | YJR105W | *ADO1* | 0.15 | Adenosine kinase |
|  | YKL211C | *TRP3* | 0.14 | Anthranilate synthase component II |
|  | YLR056W | *ERG3* | 0.20 | C-5 sterol desaturase |
|  | YML008C | *ERG6* | 0.29 | S-adenosyl-methionine delta-24-sterol-c-methyltransferase |
|  | YMR202W | *ERG2* | 0.03 | C-8 sterol isomerase |
|  | YNL280C | *ERG24* | 0.19 | C-14 sterol reductase |
|  | YMR307W | *GAS1* | 0.09 | Glycophospholipid-anchored surface glycoprotein |
|  | YPL188W | *POS5* | 0.18 | Mitochondrial NADH kinase, phosphorylates NADH, required for the response to oxidative stress |
|  | YBR249C | *ARO4* | 0.19 | 2-dehydro-3-deoxyphosphoheptonate aldolase, tyrosine-inhibited |
|  | YDR028C | *REG1* | 0.29 | Regulatory subunit for protein phosphatase Glc7p |
|  | YEL053C | *MAK10* | 0.12 | Glucose-repressible protein |
| Cell cycle and DNA processing | | | | |
|  | YML041C | *VPS71* ^b^ | 0.15 | Component of the Swr1p complex that incorporates Htz1p into chromatin, required for vacuolar protein sorting |
|  | YKR019C | *IRS4* ^b^ | 0.29 | Protein involved in regulation of phosphatidylinositol 4,5-bisphosphate concentration |
|  | YCR028C-A | *RIM1* | 0.05 | ssDNA-binding protein, mitochondrial |
|  | YNL107W | *YAF9* ^b^ | 0.12 | Component of a chromatin modifying complex |
|  | YML124C | *TUB3* | 0.30 | Alpha-3 tubulin |
|  | YAL011W | *SWC3* ^b^ | 0.24 | Component of the Swr1p complex that incorporates Htz1p into chromatin |
|  | YLR085C | *ARP6*^b^ | 0.20 | Actin-related protein |
|  | YOL012C | *HTZ1* ^b^ | 0.13 | Evolutionarily conserved member of the histone H2A F/Z family of histone variants |
|  | YBR231C | *SWC5* ^b^ | 0.09 | Component of the Swr1p complex that incorporates Htz1p into chromatin |
|  | YCL029C | *BIK1* | 0.17 | Nuclear fusion protein |
|  | YDL225W | *SHS1* | 0.23 | Septin |
|  | YDR004W | *RAD57* | 0.08 | DNA repair protein |
|  | YDR150W | *NUM1* | 0.10 | Nuclear migration protein |
|  | YDR439W | *LRS4* ^b^ | 0.19 | Involved in rDNA silencing |
|  | YDR485C | *VPS72* ^b^ | 0.25 | Component of the Swr1p complex that incorporates Htz1p into chromatin |
|  | YGR063C | *SPT4* ^b^ | 0.12 | Transcription elongation protein |
|  | YDR334W | *SWR1* ^b^ | 0.20 | DEAH-box protein, putative RNA helicase |
| Transcription | | | | |
|  | YJR063W | *RPA12* | 0.16 | DNA-directed RNA polymerase I, 13.7 KD subunit |
|  | YAL021C | *CCR4* | 0.18 | Transcriptional regulator |
|  | YNL248C | *RPA49* | 0.03 | DNA-directed RNA polymerase A (I) chain, 46 kDa |
|  | YNL025C | *SSN8* | 0.25 | DNA-directed RNA polymerase II holoenzyme and Kornberg's mediator (SRB) subcomplex subunit, cyclin C homolog |
|  | YDL213C | *NOP6* | 0.08 | Protein possible involved in rRNA processing |
| Protein synthesis | | | | |
|  | YBR191W | *RPL21A* | 0.26 | Ribosomal protein L21.e |
|  | YLR448W | *RPL6B* | 0.23 | 60S large subunit ribosomal protein |
|  | YKR057W | *RPS21A* | 0.05 | Ribosomal protein S21.e |
|  | YLR185W | *RPL37A* | 0.17 | Ribosomal protein L37.e |
|  | YDR418W | *RPL12B* | 0.14 | 60S large subunit ribosomal protein L12.e |
|  | YGR165W | *MRPS35* | 0.14 | Mitochondrial ribosomal protein, small subunit |
| Protein fate (folding, modification, destination) | | | | |
|  | YOL141W | *PPM2* | 0.25 | Putative carboxyl methyl transferase |
|  | YNL119W | *NCS2* | 0.22 | Pprotein with role in invasive growth |
|  | YKL048C | *ELM1* | 0.12 | Ser/Thr-specific protein kinase |
|  | YDR162C | *NBP2* | 0.21 | Nap1p-binding protein |
|  | YDR283C | *GCN2* | 0.13 | Ser/Thr protein kinase |
| Cellular transport, transport facilitation and transport routes | | | | |
|  | YOL158C | *ENB1* | 0.20 | Transporter of the siderophore enterobactin |
|  | YJL129C | *TRK1* | 0.16 | Potassium transporter I |
|  | YKL212W | *SAC1* ^c^ | 0.29 | Recessive suppressor of secretory defect |
|  | YKR001C | *VPS1* ^c^ | 0.14 | Member of the dynamin family of GTPases |
|  | YKR020W | *VPS51* ^c^ | 0.05 | Component of the GARP (Golgi-associated retrograde protein) complex |
|  | YLR130C | *ZRT2* | 0.23 | Zinc transporter II |
|  | YNL084C | *END3* | 0.21 | Required for endocytosis and cytoskeletal organization |
|  | YNL041C | *COG6* ^c^ | 0.30 | Conserved oligomeric golgi complex |
|  | YOR070C | *GYP1* ^c^ | 0.29 | GTPase activating protein for Ypt1p and Sec4p |
|  | YOR106W | *VAM3* ^c^ | 0.16 | Syntaxin (t-SNARE) |
|  | YBR164C | *ARL1* ^c^ | 0.26 | ADP-ribosylation factor |
|  | YDL226C | *GCS1* ^c^ | 0.19 | ADP-ribosylation factor GTPase-activating protein (ARF-GAP) |
|  | YER019C-A | *SBH2* | 0.25 | ER protein-translocation complex subunit |
|  | YGL005C | *COG7* ^c^ | 0.22 | Conserved oligomeric golgi complex |
|  | YGL054C | *ERV14* | 0.28 | ER-derived Vesicles |
|  | YGR037C | *ACB1* | 0.27 | Acyl-coenzyme-A-binding protein (diazepam binding inhibitor) |
|  | YHL031C | *GOS1* ^c^ | 0.26 | SNARE protein of Golgi compartment |
|  | YDR484W | *VPS52* ^c^ | 0.28 | Component of the GARP (Golgi-associated retrograde protein) complex |
| Others | | | | |
|  | YJR033C | *RAV1* | 0.29 | Regulator of (H^+^)-ATPase in vacuolar membrane |
|  | YBR078W | *ECM33* | 0.30 | Involved in cell wall biogenesis and architecture |
|  | YDR388W | *RVS167* | 0.11 | Reduced viability upon starvation protein |
| Unclassified proteins | | | | |
|  | YOL159C |  | 0.20 | Conserved hypothetical protein |
|  | YDR417C |  | 0.28 | Questionable protein |
|  | YML010C-B |  | 0.26 | Dubious ORF unlikely to encode a functional protein |
|  | YLR062C | *BUD28* | 0.28 | Questionable protein |
|  | YLR261C | *VPS63* | 0.20 | Questionable protein |
|  | YPL205C |  | 0.22 | Protein involved in sporulation, meiosis and nuclear division |
|  | YDR024W | *FYV1* | 0.10 | Protein of unknown function localized to cytoplasm and nucleus |
|  | YDR049W |  | 0.30 | Found in Mitochondrial Proteome |
|  | YDR136C | *VPS61* | 0.28 | Questionable protein |
|  | YGR064W |  | 0.18 | Questionable protein |

^a^Values of sensitivity were defined in methods. Sensitivity to vanillin was assessed using YPD containing 5 mM vanillin. All experiments were carried out in duplicate, and the average of the values obtained was used for the evaluation of sensitivity.

^b^These genes are involved in chromatin remodeling.

^c^These genes are involved in vesicle transport.
