# Supplementary material for: Genome-wide screening of the genes required for tolerance to vanillin, which is a potential inhibitor of bioethanol fermentation, in Saccharomyces cerevisiae
Source: Biotechnol Biofuels. 2008 Apr 15;1:3. doi: 10.1186/1754-6834-1-3 (PMC2375868; doi:10.1186/1754-6834-1-3)
Supplement: Additional file 2 — Cross-sensitivity of vanillin-sensitive mutants to various inhibitors. This list shows the cross-sensitivity of the vanillin-sensitive mutants to various fermentation inhibitors. [file 1754-6834-1-3-S2.doc]

## Additional file 2 - Cross-sensitivity of vanillin sensitive mutants to various inhibitors

|  |  |  | Sensitivity^a^ | | | | | | |
| --- | --- | --- | --- | --- | --- | --- | --- | --- | --- |
|  | ORF | Gene | VA | HB^b^ | GU^b^ | SY^b^ | FU^c^ | HMF^c^ | AA^d^ |
| Metabolism | | | | | | | | | |
|  | YML115C | *VAN1* | 0.25 | 0.13 | 0.05 | 0.11 | 0.40 | 0.06 | 0.57 |
|  | YJR105W | *ADO1* | 0.15 | 0.35 | 0.13 | 0.34 | 0.29 | 1.02 | 0.81 |
|  | YKL211C | *TRP3* | 0.14 | 0.30 | 0.02 | 0.45 | 0.13 | 0.87 | 0.10 |
|  | YLR056W | *ERG3* | 0.20 | 0.05 | 0.06 | 0.12 | 0.05 | 0.26 | 0.02 |
|  | YML008C | *ERG6* | 0.29 | 0.03 | 0.03 | 0.04 | 0.03 | 0.08 | 0.02 |
|  | YMR202W | *ERG2* | 0.03 | 0.06 | 0.03 | 0.13 | 0.05 | 0.57 | 0.02 |
|  | YNL280C | *ERG24* | 0.19 | 0.04 | 0.02 | 0.10 | 0.09 | 0.27 | 0.01 |
|  | YMR307W | *GAS1* | 0.09 | 1.25 | 0.32 | 1.01 | 0.32 | 0.98 | 0.76 |
|  | YPL188W | *POS5* | 0.18 | 0.49 | 0.32 | 0.36 | 0.51 | 1.41 | 0.35 |
|  | YBR249C | *ARO4* | 0.19 | 0.58 | 0.15 | 0.70 | 0.11 | 0.29 | 0.46 |
|  | YDR028C | *REG1* | 0.29 | 0.06 | 0.03 | 0.16 | 0.03 | 0.66 | 0.02 |
|  | YEL053C | *MAK10* | 0.12 | 0.36 | 0.56 | 0.37 | 0.60 | 1.07 | 0.63 |
| Cell cycle and DNA processing | | | | | | | | | |
|  | YML041C | *VPS71* | 0.15 | 0.48 | 0.29 | 0.45 | 0.23 | 0.77 | 0.15 |
|  | YKR019C | *IRS4* | 0.29 | 0.28 | 0.36 | 0.34 | 0.43 | 1.02 | 0.65 |
|  | YCR028C-A | *RIM1* | 0.05 | 0.01 | 0.01 | 0.09 | 0.01 | 0.04 | 0.01 |
|  | YNL107W | *YAF9* | 0.12 | 0.20 | 0.03 | 0.14 | 0.13 | 0.22 | 0.09 |
|  | YML124C | *TUB3* | 0.30 | 0.29 | 0.45 | 0.61 | 0.60 | 0.89 | 0.54 |
|  | YAL011W | *SWC3* | 0.24 | 0.19 | 0.05 | 0.08 | 0.09 | 0.47 | 0.02 |
|  | YLR085C | *ARP6* | 0.20 | 0.53 | 0.67 | 0.46 | 0.64 | 0.33 | 0.58 |
|  | YOL012C | *HTZ1* | 0.13 | 0.32 | 0.17 | 0.26 | 0.28 | 0.33 | 0.12 |
|  | YBR231C | *SWC5* | 0.09 | 0.67 | 0.31 | 0.47 | 0.24 | 0.36 | 0.19 |
|  | YCL029C | *BIK1* | 0.17 | 0.24 | 0.10 | 0.45 | 0.18 | 0.58 | 0.46 |
|  | YDL225W | *SHS1* | 0.23 | 0.67 | 0.08 | 0.38 | 0.31 | 0.64 | 0.06 |
|  | YDR004W | *RAD57* | 0.08 | 0.84 | 0.12 | 0.62 | 0.28 | 0.98 | 0.55 |
|  | YDR150W | *NUM1* | 0.10 | 0.72 | 0.38 | 0.56 | 0.38 | 0.42 | 0.43 |
|  | YDR439W | *LRS4* | 0.19 | 0.77 | 0.22 | 0.69 | 0.24 | 0.93 | 0.38 |
|  | YDR485C | *VPS72* | 0.25 | 0.60 | 0.18 | 0.44 | 0.29 | 0.61 | 0.14 |
|  | YGR063C | *SPT4* | 0.12 | 0.88 | 0.55 | 0.83 | 0.56 | 0.71 | 0.57 |
|  | YDR334W | *SWR1* | 0.20 | 0.36 | 0.32 | 0.40 | 0.42 | 0.37 | 0.28 |
| Transcription | | | | | | | | | |
|  | YJR063W | *RPA12* | 0.16 | 1.10 | 0.94 | 0.98 | 0.98 | 1.30 | 0.90 |
|  | YAL021C | *CCR4* | 0.18 | 0.45 | 0.27 | 0.23 | 0.59 | 1.41 | 0.19 |
|  | YNL248C | *RPA49* | 0.03 | 0.49 | 0.03 | 0.58 | 0.17 | 0.53 | 0.53 |
|  | YNL025C | *SSN8* | 0.25 | 0.23 | 0.03 | 0.50 | 0.06 | 0.26 | 0.87 |
|  | YDL213C | *NOP6* | 0.08 | 0.46 | 0.15 | 0.33 | 0.28 | 0.47 | 0.18 |
| Protein synthesis | | | | | | | | | |
|  | YBR191W | *RPL21A* | 0.26 | 0.32 | 0.61 | 0.65 | 0.45 | 0.72 | 0.43 |
|  | YLR448W | *RPL6B* | 0.23 | 0.56 | 0.36 | 0.79 | 0.49 | 0.69 | 0.83 |
|  | YKR057W | *RPS21A* | 0.05 | 0.57 | 0.16 | 0.61 | 0.34 | 0.63 | 0.46 |
|  | YLR185W | *RPL37A* | 0.17 | 0.28 | 0.25 | 0.41 | 0.39 | 0.35 | 0.78 |
|  | YDR418W | *RPL12B* | 0.14 | 0.35 | 0.03 | 0.45 | 0.04 | 0.17 | 0.09 |
|  | YGR165W | *MRPS35* | 0.14 | 0.46 | 0.57 | 0.34 | 0.72 | 0.45 | 0.77 |
| Protein fate (folding, modification, destination) | | | | | | | | | |
|  | YOL141W | *PPM2* | 0.25 | 0.75 | 0.35 | 0.62 | 0.45 | 0.44 | 0.38 |
|  | YNL119W | *NCS2* | 0.22 | 0.55 | 0.25 | 0.64 | 0.46 | 1.49 | 0.75 |
|  | YKL048C | *ELM1* | 0.12 | 0.33 | 0.10 | 0.45 | 0.10 | 0.59 | 0.46 |
|  | YDR162C | *NBP2* | 0.21 | 0.05 | 0.42 | 0.24 | 0.35 | 0.52 | 0.56 |
|  | YDR283C | *GCN2* | 0.13 | 0.70 | 0.78 | 0.44 | 0.55 | 0.56 | 0.87 |
| Cellular transport, transport facilitation and transport routes | | | | | | | | | |
|  | YOL158C | *ENB1* | 0.20 | 0.89 | 0.23 | 0.87 | 0.39 | 0.80 | 0.66 |
|  | YJL129C | *TRK1* | 0.16 | 0.85 | 0.36 | 0.85 | 0.58 | 0.95 | 0.59 |
|  | YKL212W | *SAC1* | 0.29 | 0.16 | 0.02 | 0.21 | 0.12 | 0.25 | 0.01 |
|  | YKR001C | *VPS1* | 0.14 | 0.09 | 0.34 | 0.14 | 0.52 | 0.76 | 0.02 |
|  | YKR020W | *VPS51* | 0.05 | 0.07 | 0.17 | 0.13 | 0.14 | 0.32 | 0.05 |
|  | YLR130C | *ZRT2* | 0.23 | 0.71 | 0.25 | 0.69 | 0.43 | 0.43 | 0.80 |
|  | YNL084C | *END3* | 0.21 | 0.44 | 0.40 | 0.32 | 0.30 | 0.42 | 0.10 |
|  | YNL041C | *COG6* | 0.30 | 0.18 | 0.43 | 0.39 | 0.65 | 0.60 | 0.22 |
|  | YOR070C | *GYP1* | 0.29 | 0.05 | 0.34 | 0.41 | 0.66 | 0.86 | 0.60 |
|  | YOR106W | *VAM3* | 0.16 | 0.25 | 1.14 | 0.32 | 1.08 | 0.79 | 0.84 |
|  | YBR164C | *ARL1* | 0.26 | 0.13 | 0.71 | 0.14 | 0.41 | 0.63 | 0.17 |
|  | YDL226C | *GCS1* | 0.19 | 0.53 | 0.56 | 0.74 | 0.63 | 0.84 | 0.39 |
|  | YER019C-A | *SBH2* | 0.25 | 0.95 | 0.30 | 0.97 | 0.47 | 1.20 | 0.95 |
|  | YGL005C | *COG7* | 0.22 | 0.29 | 0.27 | 0.54 | 0.30 | 0.74 | 0.31 |
|  | YGL054C | *ERV14* | 0.28 | 0.45 | 0.05 | 0.64 | 0.19 | 0.80 | 0.30 |
|  | YGR037C | *ACB1* | 0.27 | 0.65 | 1.05 | 0.67 | 0.77 | 0.47 | 0.22 |
|  | YHL031C | *GOS1* | 0.26 | 0.16 | 1.03 | 0.29 | 0.95 | 0.81 | 0.91 |
|  | YDR484W | *VPS52* | 0.28 | 0.07 | 0.20 | 0.19 | 0.18 | 0.38 | 0.12 |
| Other | | | | | | | | | |
|  | YJR033C | *RAV1* | 0.29 | 0.25 | 0.50 | 0.34 | 0.57 | 1.33 | 0.14 |
|  | YBR078W | *ECM33* | 0.30 | 0.26 | 0.10 | 0.33 | 0.44 | 0.64 | 0.01 |
|  | YDR388W | *RVS167* | 0.11 | 0.03 | 0.06 | 0.20 | 0.21 | 0.47 | 0.25 |
| Unclassified proteins | | | | | | | | | |
|  | YOL159C |  | 0.20 | 0.62 | 0.25 | 0.66 | 0.58 | 1.02 | 0.55 |
|  | YDR417C |  | 0.28 | 0.19 | 0.03 | 0.33 | 0.06 | 0.26 | 0.24 |
|  | YML010C-B |  | 0.26 | 0.59 | 0.86 | 0.88 | 0.86 | 1.07 | 0.96 |
|  | YLR062C | *BUD28* | 0.28 | 0.30 | 0.38 | 0.23 | 0.62 | 0.28 | 0.45 |
|  | YLR261C | *VPS63* | 0.20 | 0.19 | 0.47 | 0.35 | 0.72 | 0.85 | 0.70 |
|  | YPL205C |  | 0.22 | 0.36 | 1.18 | 0.39 | 1.19 | 0.81 | 0.35 |
|  | YDR024W | *FYV1* | 0.10 | 0.63 | 0.10 | 0.48 | 0.42 | 0.83 | 0.36 |
|  | YDR049W |  | 0.30 | 0.19 | 0.29 | 0.30 | 0.11 | 0.04 | 0.02 |
|  | YDR136C | *VPS61* | 0.28 | 0.15 | 0.87 | 0.31 | 0.22 | 0.33 | 0.10 |
|  | YGR064W |  | 0.18 | 0.06 | 0.28 | 0.15 | 0.61 | 0.25 | 0.15 |

^a^Values of sensitivity were defined in methods; values < 0.3 are shaded. All experiments were carried out in duplicate, and the average of the values obtained was used for the evaluation of sensitivity.

^b^Sensitivity to phenolic compounds was assessed using YPD containing 7 mM HB, 7 mM syringaldehyde or 16 mM guaiacol.

^c^Sensitivity to furan derivatives was assessed using YPD containing 11 mM furfural or 15 mM HMF.

^d^Sensitivity to weak acid was assessed using YPD containing 70 mM acetic acid.

VA, vanillin; HB, 4-hydroxybenzoic acid; GU, guaiacol; SY, syringaldehyde; FU, furfural; HMF, 5-hydroxymethylfurfural; AA, acetic acid.
